# Rare Presentation of Fever of Unknown Origin: A Diagnostic Challenge

**DOI:** 10.7759/cureus.96341

**Published:** 2025-11-07

**Authors:** Tanya Kondolay, Rojina Samifanni, Guo Wang, Jerry Abraham, Muhammad A Shahzad

**Affiliations:** 1 Preventive Medicine, Windsor University School of Medicine in St. Kitts, Cayon, KNA; 2 Internal Medicine, St. George's University School of Medicine, St. George's, GRD; 3 Obstetrics and Gynecology, St. George's University School of Medicine, St. George's, GRD; 4 Internal Medicine, AMITA Health Adventist Medical Center GlenOaks, Glendale Heights, USA

**Keywords:** candiduria, diagnostic dilemma, fever of unknown origin, lymphadenopathy, pelvic inflammatory disease, pelvic pain, postmenopause, recurrent uti

## Abstract

We present the case of a 49-year-old postmenopausal female patient with recurrent urinary tract infections, chronic pelvic pain, and persistent systemic symptoms, including fever, hypotension, and leukocytosis, admitted five times over a three-month period. Despite extensive antimicrobial treatment, imaging revealed retroperitoneal and gastrohepatic lymphadenopathy with pancreatic fat stranding, raising suspicion for malignancy or disseminated fungal infection. The patient’s complex presentation, occupational exposure, past gynecologic procedures, and linguistic barriers compounded the diagnostic challenge. This case met the classic criteria for fever of unknown origin (FUO) and emphasizes the need for a multidisciplinary approach to rare pelvic infection presentations in postmenopausal women.

## Introduction

Fever of unknown origin (FUO) was first defined by Petersdorf and Beeson in 1961 as a body temperature >38.3°C on at least three occasions, persisting for more than three weeks, and remaining undiagnosed after one week of inpatient evaluation [1]. While many cases resolve spontaneously, others are linked to serious conditions such as infection, malignancy, or autoimmune disease [2]. Hematologic malignancies are identified in approximately 10-15% of FUO cases and carry higher mortality [3]. Rarely, behavioral contributors such as factitious fever must also be considered.

We present the case of a postmenopausal woman who met FUO criteria following multiple admissions for systemic illness, including septic shock. Despite an extensive workup, no definitive etiology was identified, emphasizing the diagnostic uncertainty inherent in FUO.

## Case presentation

A 49-year-old Spanish-speaking woman presented with repeated episodes of fever, hypotension, and pelvic pain on December 8, 2024. She had a history of recurrent urinary tract infection (UTI) since 2016, early menopause at age 38, and uterine fibroid embolization. She also had a remote chlamydia infection in 2004, treated successfully. Past surgical history included tubal ligation and cholecystectomy. She reported a monogamous relationship since 2004. Between October and December 2024, the patient was hospitalized five times, including one ICU admission for septic shock requiring vasopressor support.

Her symptoms included fever, nausea, vomiting, foul-smelling white vaginal discharge, vaginal itching, left lower quadrant (LLQ) abdominal pain, and concurrent left flank/back pain. She reported that LLQ abdominal pain was consistently accompanied by discomfort in the corresponding area of her back.

On examination, she was febrile with LLQ tenderness and guarding, along with focal vaginal wall tenderness. No external lesions or discharge were observed. No palpable lymphadenopathy was present. Laboratory findings demonstrated recurrent neutrophilia, monocytosis, and elevated C-reactive protein (CRP) (Table 1). Urinalysis repeatedly demonstrated yeast, while blood cultures and STI screening remained negative. Both random and fasting plasma glucose levels were obtained, as summarized in Table 2. HbA1c was not assessed, given that serial fasting glucose values consistently remained within normal limits. The transient elevations observed in random glucose values were considered likely attributable to physiologic stress related to infection and fever, or to post-prandial sampling.

**Table 1:** Inflammatory and infectious marker trends from prior hospital admissions to the current presentation CRP: C-reactive protein; +: positive result, ANA: antinuclear antibody

Parameters	December 8	December 6	December 4	December 1	November 30	November 28	November 20	November 13	November 5	November 3	October 21	October 20	Reference Range
Neutrophils	10.74	7.16	13.8	-	9.19	22.5	9.12	-	-	10.64	12.35	14.6	1.70-6.70 10*3/uL
Monocytes	0.86	0.63	0.76	-	0.76	1.22	0.66	-	-	0.64	0.87	0.44	0.15-0.58 10*3uL
Lactic Acid	-	-	-	-	-	2.3	-	4.3	2.63	2.5	-	-	0.5 - 2.0 mmol/L
CRP	-	-	-	-	-	19.4	-	-	-	-	-	-	< 1.0 mg/L
ANA IgG	-	-	-	-	-	-	-	Trace	-	-	-	-	-
Clostridium difficile	-	-	-	+	-	-	-	-	-	-	-	-	

**Table 2:** Blood glucose levels

	December 5	December 4	December 1	November 30	November 28	November 20	November 16	November 15	November 14	November 13	November 11	November 5	November 3	October 21	October 20	Reference Range
Glucose	-	89	144	102	111	104	-	107	171	125	-	122	120	121	157	70-99 mg/dL
Fasting Blood Glucose	93	-	-	-	-	-	98	-	88	98	98	-	-	-	-	>100 mg/dL

**Figure 1:**
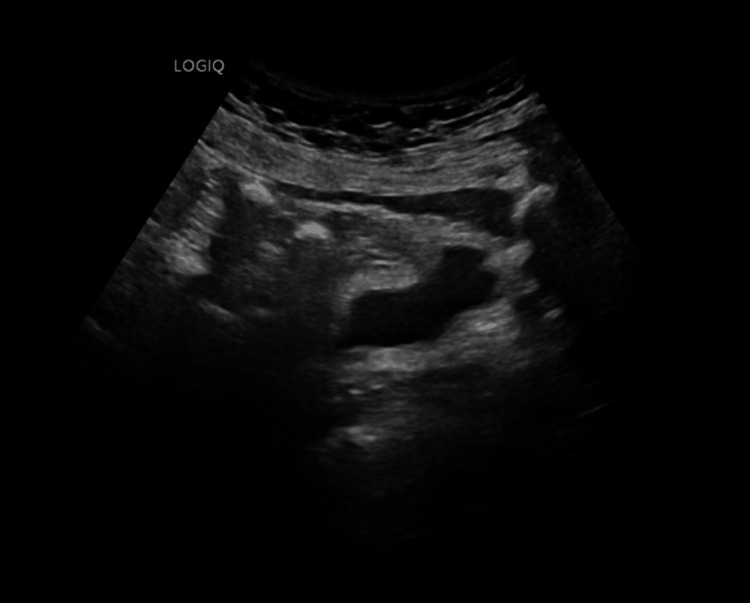
Pelvic ultrasound showing a small, heavily calcified uterus with a visible right ovary.

**Figure 2:**
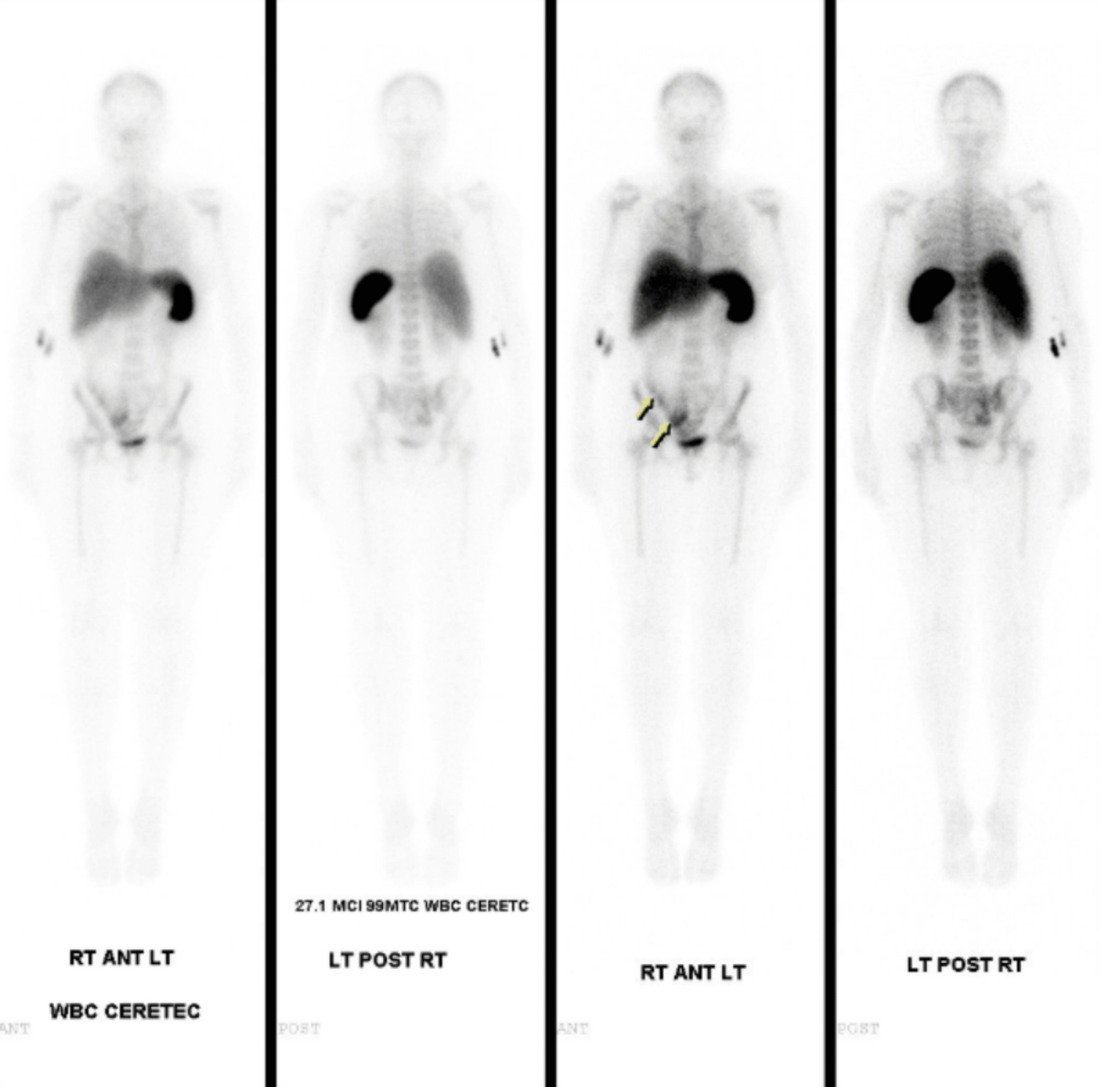
Whole-body nuclear medicine white cell scan (99mTc-Ceretec) demonstrating tubular and linear radiopharmaceutical uptake in the right lower quadrant, suggestive of an infectious or inflammatory process involving the cecum/ascending colon with possible distal small bowel involvement.

Imaging included pelvic ultrasound, which demonstrated a small, heavily calcified uterus with a visible right ovary (Figure 1). CT imaging of the abdomen and pelvis revealed retroperitoneal and gastrohepatic lymphadenopathy with peripancreatic fat stranding. A nuclear medicine white cell scan using 99mTc-Ceretec showed tubular and linear radiopharmaceutical uptake in the right lower quadrant, suggestive of an infectious or inflammatory process involving the cecum/ascending colon with possible distal small bowel involvement (Figure 2). The corresponding CT during the same admission did not demonstrate definitive inflammatory or structural abnormalities, underscoring the discordance and diagnostic uncertainty.

CT-guided lymph node biopsy revealed reactive changes, while bone marrow biopsy demonstrated normocellular marrow without evidence of hematologic malignancy. An exploratory laparotomy performed during her fifth admission revealed no acute intra-abdominal pathology.

She was treated with multiple courses of empiric antimicrobials (meropenem, vancomycin) and antifungals (fluconazole, micafungin), with only transient symptomatic improvement. Multidisciplinary input was obtained from infectious disease, gynecology, oncology, surgery, and cardiology.

The patient required interpreter services due to limited English proficiency, and inconsistencies were noted in her reporting of symptoms between providers. She repeatedly requested intravenous Dilaudid (hydromorphone hydrochloride), describing it as the only effective pain relief. Factitious fever was therefore considered as part of the differential diagnosis. Following her final discharge, she was scheduled for outpatient follow-up but was subsequently lost to follow-up.

## Discussion

This case underscores the diagnostic ambiguity that arises when postmenopausal infections present with systemic signs and atypical imaging. Although pelvic inflammatory disease (PID) is rare in postmenopausal women [4], it may persist due to chronic infections or anatomic changes from prior gynecologic procedures, including tubal ligation and uterine fibroid embolization. Estrogen deficiency in postmenopause is also associated with urogenital atrophy, which can predispose to infection and recurrent pelvic symptoms.

The detection of yeast in urine alongside systemic illness raised concern for fungal UTI or disseminated candidiasis. While candiduria is often benign, it may be clinically significant [5] in patients with underlying structural abnormalities, prior urologic procedures, or immunosuppression. In this patient, recurrent candiduria in the context of fever, leukocytosis, and hypotension justified empiric antifungal therapy. Although blood cultures remained negative, published literature emphasizes that candiduria with systemic features warrants close evaluation and, in some cases, empirical antifungal management.

Radiologic findings, particularly retroperitoneal and gastrohepatic lymphadenopathy with peripancreatic fat stranding, heightened concern for hematologic or metastatic malignancy. Lymph node biopsy was therefore pursued, consistent with current guidance, as hematologic malignancies account for approximately 10-15% of FUO cases and carry higher mortality [6]. Despite this, both the lymph node biopsy and bone marrow aspirate were nondiagnostic, highlighting the limitations of invasive testing in complex FUO cases.

Further diagnostic complexity arose from the nuclear medicine white cell scan, which demonstrated tubular and linear radiopharmaceutical uptake in the right lower quadrant. This pattern suggested an infectious or inflammatory process involving the cecum or distal small bowel. However, exploratory laparotomy revealed no acute intra-abdominal pathology, reinforcing the classification of this case as true FUO.

Beyond medical considerations, psychosocial and environmental factors played an important role. Communication barriers due to limited English proficiency affected continuity of care and reliability of the history. Despite interpreter support, discrepancies were noted in her accounts of symptoms and pain between different providers and she repeatedly request for intravenous Dilaudid, describing it as the only effective pain relief. This behavior raised concern for possible factitious disorder or somatization, both of which are rare but recognized causes of FUO.

Occupational exposure also warranted consideration. The patient worked long shifts in a plastic manufacturing facility, potentially exposing her to chemical agents [7] known to influence immune and inflammatory responses. While a direct causal relationship could not be established, the contribution of environmental exposure to unexplained systemic illness should not be overlooked.

Ultimately, this case exemplifies the need for an interdisciplinary and culturally competent approach to FUO. Even when conventional diagnostics remain inconclusive, thoughtful escalation, patient-centered investigation, and clinical restraint are critical to avoid unnecessary harm. This diagnostic process may also be shaped by evolving evidence on changes in the vaginal microbiome [8] and the high prevalence of recurrent UTIs in postmenopausal women [9]. Taken together, these factors illustrate the nuanced interplay of infection, malignancy, environment, and behavior in the evaluation of FUO.

## Conclusions

This case illustrates the complexity of FUO in a postmenopausal woman with recurrent pelvic pain and systemic illness, where overlapping infectious, oncologic, and behavioral factors complicated diagnosis. Despite extensive evaluation, including advanced imaging, biopsy, and exploratory laparotomy, no definitive etiology was identified. All suspected infectious, inflammatory, and environmental etiologies remained unconfirmed, and exploratory laparotomy was pursued based on persistent clinical concern rather than a single definitive localizing study.

Such cases highlight the importance of maintaining a broad differential, incorporating both organic and behavioral causes, and recognizing the potential influence of environmental exposures. Ultimately, effective management of FUO requires a multidisciplinary, culturally competent approach that balances the pursuit of diagnosis with restraint to avoid unnecessary invasive testing, ensuring patient safety while preserving diagnostic rigor.
